# Changes in Expression of Genes Regulating Airway Inflammation Following a High-Fat Mixed Meal in Asthmatics

**DOI:** 10.3390/nu8010030

**Published:** 2016-01-07

**Authors:** Qian Li, Katherine J. Baines, Peter G. Gibson, Lisa G. Wood

**Affiliations:** 1 Centre for Asthma and Respiratory Diseases, Hunter Medical Research Institute, University of Newcastle, NSW 2308, Australia; qli3@uon.edu.au (Q.L.); katherine.baines@newcastle.edu.au (K.J.B.); Peter.gibson@hnehealth.nsw.gov.au (P.G.G.); 2 Department of Respiratory and Sleep Medicine, John Hunter Hospital, Newcastle, NSW 2305, Australia

**Keywords:** asthma, inflammation, fatty acids

## Abstract

Consumption of a high fat meal can increase neutrophilic airway inflammation in asthma subjects. This study investigates the molecular mechanisms driving airway neutrophilia following a high fat meal in asthmatics. Subjects with asthma (*n* = 11) and healthy controls (*n* = 8) consumed a high-fat/energy meal, containing total energy (TE) of 3846 kJ and 48 g of total fat (20.5 g saturated). Sputum was induced at 0 and 4 h, and gene expression was examined by microarray and quantitative real-time PCR (qPCR). Following the high fat dietary challenge, 168 entities were significantly differentially expressed greater than >1.5 fold in subjects with asthma, whereas, in healthy controls, only 14 entities were differentially expressed. Of the 168 genes that were changed in asthma, several biological processes were overrepresented, with 25 genes involved in “immune system processes”. qPCR confirmed that S100P, S100A16, MAL and MUC1 were significantly increased in the asthma group post-meal. We also observed a strong correlation and a moderate correlation between the change in NLRP12 and S100A16 gene expression at 4 h compared to baseline, and the change in total and saturated non-esterified plasma fatty acid levels at 2 h compared to baseline. In summary, our data identifies differences in inflammatory gene expression that may contribute to increased airway neutrophilia following a high fat meal in subjects with asthma and may provide useful therapeutic targets for immunomodulation. This may be particularly relevant to obese asthmatics, who are habitually consuming diets with a high fat content.

## 1. Introduction

Asthma is a significant global disease which is a common source of morbidity and a significant cause of preventable mortality especially in Western countries, such as USA, Great Britain, Canada, Australia and New Zealand [1]. Asthma is a chronic inflammatory disease of the airways [2], and the nature of inflammation in asthma is heterogeneous. While allergen-induced T-helper type 2 immune activation leads to airway eosinophilia, asthma can also involve innate immune dysfunction, with increased airway neutrophilia, sputum IL-8 and gene expression of TLR2 and TLR4 [2]. Airway neutrophils are clinically important, as neutrophil counts increase with asthma severity [3] and sputum neutrophils negatively correlate with lung function and airflow obstruction in asthma [4]. Thus, stimuli that increase airway neutrophilia may contribute to asthma pathophysiology.

As asthma prevalence has increased in recent decades and has also been shown to develop in susceptible individuals who have migrated from a developing to a Westernized country [5,6,7], non-genetic, environmental factors, such as dietary intake, appear likely to be contributing to asthma onset and development [8]. Dietary fat is an essential macronutrient, necessary for a variety of body functions such as formation and functioning of cell membranes. However, recently, dietary fatty acids have been linked to inflammation, with various mechanisms being proposed. For example, fatty acids induce innate immune responses through activation of Toll-like receptor (TLR)-4 and initiation of an NF-κB-mediated inflammatory cascade [9]. Dietary fat has been linked to asthma in several observational studies, with total fat intake and plasma triglyceride levels being associated with airway hyperresponsiveness [10], asthma risk [11] and adult-onset wheeze [12]. We previously conducted the first study to directly examine the effect of dietary fat on systemic and airway inflammation in asthma. We reported that subjects who consumed a high fat, high energy mixed meal, had an increase in sputum %neutrophils and TLR4 mRNA expression at 4 h, compared to subjects who consumed a low fat, low energy meal. Furthermore, changes in sputum %neutrophils correlated with changes in plasma fatty acids [13].

While our previous investigations have established a role for TLR4 signalling in response to the high fat challenge, it is likely that other inflammatory pathways are also important. For example, saturated fatty acids can activate nucleotide binding oligomerization domain (NOD)-containing proteins, resulting in modulation of NF-κB and CXCL8 gene expression [14]. The study of gene expression using microarray technology (transcriptomics) can be used to examine changes in gene expression of the whole genome, which provides a sound basis for mechanistic studies. The aim of this study was to use microarray techniques to examine the molecular mechanisms of fat-induced airway inflammation in asthma.

## 2. Methods

### 2.1. Subject Recruitment

Subjects with stable asthma were recruited via ambulatory care clinics at the John Hunter Hospital or from existing study databases. Asthma was defined by clinical history and airway hyper responsiveness (AHR) to hypertonic saline (4.5%) greater than 15% from baseline. Asthma severity was classified using global initiative for asthma (GINA) criteria. Stable asthma was defined as no exacerbation, respiratory tract infection or oral corticosteroids in the past 4 weeks. Atopy was assessed using skin prick allergy testing. Healthy controls were recruited from existing study databases, who had no respiratory symptoms, had never had a doctor’s diagnosis of asthma, had normal lung function without airway hyper responsiveness and were steroid naïve*.* All subjects were non-smokers. Written informed consent was obtained from all subjects and the study was approved by the Hunter New England Human Research Ethics Committee. A subset of data collected from these subjects has previously been reported [13].

### 2.2. Study Design

Subjects with asthma (*n* = 11) and healthy controls (*n* = 8) were provided with a high-fat, high energy meal (2 breakfast burgers and 2 hash browns). The high-fat meal contained total energy (TE) of 3846 kJ and consisted of 79 g (33% of TE) carbohydrate, 39 g (18% of TE) protein, and 48 g (49% of TE) total fat, including 20.5 g (21% of TE) saturated fat. At baseline (time = 0), sputum was induced, and the study meal was consumed within 15 min. Sputum was induced again at 4 h. Blood was collected at baseline 2, 3 and 4 h after the meal. To investigate the effects of the high fat meal on airway inflammation, sputum gene expression was examined by microarray and confirmed using quantitative real-time PCR (qPCR) in a subset of subjects (asthmatics, *n* = 5; healthy controls, *n* = 3), and qPCR was subsequently performed on all asthmatic samples (*n* = 11).

### 2.3. Sample Collection and Processing

Spirometry (forced expiratory volume in 1 s (FEV_1_) and forced vital capacity (FVC)) and sputum induction with hypertonic saline (4.5%) was performed as previously described [15]. Lower respiratory sputum portions were selected and dispersed using dithiothreitol [15]. Total cell counts and cell viability were performed by haemocytometer and cytospins used for differential cell counts. For gene expression analysis, 100 μL of selected sputum plugs were added to Buffer RLT (Qiagen, Hilden, Germany) and stored at −80 °C until RNA extraction. Peripheral blood was collected in EDTA tubes and centrifuged at 4 °C, 3000 *g*, for 10 min. Plasma was separated and stored at −80 °C for analysis of non-esterified fatty acid concentrations using gas chromatography.

### 2.4. Whole Genome Gene Expression Microarrays

Whole genome gene expression analysis was performed in paired sputum samples. RNA was extracted from induced sputum using the RNeasy Mini Kit (Qiagen, Hilden, Germany) and quantitated using the Quant-iT RiboGreen RNA Quantitation Assay Kit (Molecular Probes Inc., Life Technologies, Carlsbad, CA, USA), where fluorescence was measured at wavelengths 485 nm (excitation) and 520 nm (emission) (FLUOstar Optima, BMG Labtech, VIC, Australia). A total of 500 ng of sputum RNA was amplified and labelled with biotin-uridine triphosphate using the Total Prep RNA amplification kit as per manufacturer’s instructions (Life Technologies). 750 ng cRNA was hybridised to Illumina Sentrix HumanRef-8 V3 Expression BeadChips (Illumina, San Diego, CA, USA) using standard protocols. Each BeadChip measured the expression of 24,354 genes and was scanned using the Illumina Bead Station and captured using BeadScan 3.5.11 (Illumina). The microarray primary data used in this study are available at the national center for biotechnology information (NCBI) Gene Expression Omnibus (http://www.ncbi.nlm.nih.gov/geo/) under accession number GSE74075.

### 2.5. Quantitative Real-Time PCR (qPCR)

RNA (200 ng) was reverse-transcribed to cDNA using the high-capacity cDNA reverse transcription kit (Applied Biosystems, Foster City, CA, USA), after DNase I digestion (Life nologies Carlsbad, CA, USA) following manufacturer’s instructions. Taqman PCR primers and probes for MUC1, S100P, S100A16, NLRP12 and MAL were obtained as proprietary pre-optimised reagents (Applied Biosystems, Life Technologies). PCR primers and probes were combined with Taqman gene expression master mix in duplicate singleplex real time PCR reactions (7500 Real-Time PCR System, Applied Biosystems). Relative quantitation of mRNA expression was determined using the 2^−∆∆Ct^ calculation relative to the housekeeping genes (18S rRNA) and the baseline mean of the healthy group [16].

### 2.6. Non-Esterified Plasma Fatty Acid Concentrations

Non-esterified fatty acids (NEFA) were measured using gas chromatography. C13:0 and C19:0 (0.02 g/L) were added to plasma as internal standards. Fatty acids were methylated by adding methanol/ acetyl chloride, then heating the sample to 26 °C for 45 min. After cooling, the reaction was stopped by adding K_2_CO_3_, hexane was added, and then the sample was centrifuged at 3000 *g* for 10 min. The upper toluene layer was used for gas chromatography analysis of the fatty acid methyl esters by use of a 30 m × 0.25 mm fused carbon-silica column (DB-225) coated with cyanopropylphenyl (J&W Scientific, Folsom, CA, USA) and a flame ionization detector. Sample fatty acid methyl ester peaks were identified by comparing their retention times with those of a standard mixture of fatty acid methyl esters and were quantified by using a Hewlett Packard 6890 series gas chromatograph with Chemstations software (version A.04.02; Palo Alto, CA, USA) for gas chromatographic analysis.

### 2.7. Statistical Analysis

The clinical characteristics, sputum cell counts and qPCR results were analysed using Stata 11 (Stata Corp, College Station, TX, USA). Nonparametric data were reported as median (Q1, Q3). Statistical comparisons were conducted using a *t*-test or Wilcoxon rank test for quantitative data, and Chi-squared test or Fisher’s exact test for frequency data. *p* < 0.05 was considered to be significant. Whole genome gene expression data were exported to GeneSpring GX12 (Agilent Technologies) using Illumina’s Bead Studio, log transformed and baseline converted to the median of all samples. Differential gene expression after consumption of the fatty meal was analysed using a paired *t*-test, and then filtered on a volcano plot using the following restrictions. Genes were considered significant when (1) the gene was detected as present or marginal in all sputum samples at baseline; (2) the extent of the difference in gene expression was statistically significant (*p* < 0.05 paired *t*-test adjusted for multiple comparisons using the Benjamini Hochberg method); and (3) the difference in expression was greater than 1.5 fold. The protein annotation through evolutionary relationship (PANTHER) classification system (http://www.pantherdb.org/) [17], was used to investigate the biological processes (GO-Slim) that were overrepresented in our differentially expressed gene set.

## 3. Results

### 3.1. Clinical Characteristics of Asthmatics and Healthy Controls Group

The clinical characteristics of 11 asthmatics and 8 healthy controls are summarized in Table 1. The mean (SD) age of asthmatics was 48.5 (18.7) years and healthy controls was 49.4 (18.8) years. There were six males and five females in the asthma group, and six males and two females in the healthy control group. The healthy group had significantly more ex-smokers, were less atopic and had a higher FEV_1_/FVC%. There were no significant differences in BMI, FEV_1_% or FVC%. The higher total cell count, %neutrophils and %eosinophils in the asthmatic group were not statistically significant.

**Table 1:** Clinical characteristics of asthmatics and healthy controls.

	Asthma	Healthy	*p*-Value
*N*	11	8	NA
Age	48.5 (18.7)	49.4 (18.8)	0.925
Gender (M|F)	6|5	6|2	0.361
BMI, mean (SD)	30.6 (5.1)	29.0 (5.5)	0.514
Smoking, ex|never	1|10	5|3	0.013
Atopy, *n* (%)	9 (82)	2 (25)	0.013
FEV1%, mean (SD)	83.9 (25.3)	101.8 (13.8)	0.088
FVC%, mean (SD)	100.4 (21.1)	104.8 (16.8)	0.626
FEV1/FVC%, mean (SD)	67.3 (11.5)	78.8 (6.6)	0.021
Total cell count (10^6^/mL), median (Q1,Q3)	4.8 (3.9–6.1)	3.6 (1.2–8.2)	0.457
Neutrophils%, median (Q1,Q3)	45.5 (23.8–68)	33.8 (22.8–50.1)	0.65
Eosinophils%, median (Q1,Q3)	2.8 (0.3–7.8)	0.1 (0–2.8)	0.141
SNEFA (mg/L), mean (SD)	156.7 (56.5)	123.3 (54.9)	0.215
Total NEFA (mg/L), mean (SD)	449.5 (170.3)	376.5 (185.2)	0.386

NEFA, non-esterified fatty acids; SNEFA, saturated non-esterified fatty acids; BMI, body mass index; FEV1%, forced expiratory volume in 1 s as percentage of predicted value; FVC%, forced vital capacity as percentage of predicted value.

### 3.2. Effects of a High Fat Challenge on Sputum Gene Expression

A gene was considered expressed in sputum if it was flagged as present or marginal in all sputum samples at baseline. This resulted in 10,850 out of 24,526 entities being expressed (44.2%). Following the high fat dietary challenge, the expressions of 672 entities were significantly different at 4 h in asthma. A volcano plot analysis was used to further refine this list and select those genes that were both significantly different (adjusted *p* < 0.05) and had a fold change of >1.5, which resulted in 168 entities changed in asthma. Out of these 168 entities, 68 were increased in expression and 100 were decreased in expression after the fat challenge. Biological processes that were overrepresented in this gene list were determined using PANTHER (Go-slim) and are shown in Table 2. The top category that was overrepresented was immune system processes which contained 25 genes altered with the high fat diet (*p* = 0.0003, Table 3).

**Table 2:** Panther Go-slim Biological Processes which were overrepresented in sputum cells from asthmatics, at 4 h after a high fat meal compared with baseline.

GO-Slim Category	Genes in the Reference List (*n* =)	Genes in the Experiment List (*n* =)	Genes Expected (*n* =)	Fold Enrichment	*p*-Value
immune system process	1733	25	11.92	2.1	0.0003
cellular defense response	387	9	2.66	3.38	0.002
cell adhesion	890	14	6.12	2.29	0.004
biological adhesion	890	14	6.12	2.29	0.004
metabolic process	8613	75	59.25	1.27	0.006

In healthy controls, following the high fat dietary challenge, the expressions of 343 entities were significantly different at 4 h. A volcano plot analysis was used to further refine this list and select those genes that were both significantly different (adjusted *p* < 0.05) and had a fold change of >1.5, which resulted in 14 entities changed in the healthy group. Out of these 14 entities, 13 were increased in expression and one was decreased in expression after the fat challenge (Table 4).

### 3.3. Quantitative Real Time PCR (qPCR) Validation Of Sputum Results

**Figure 1:**
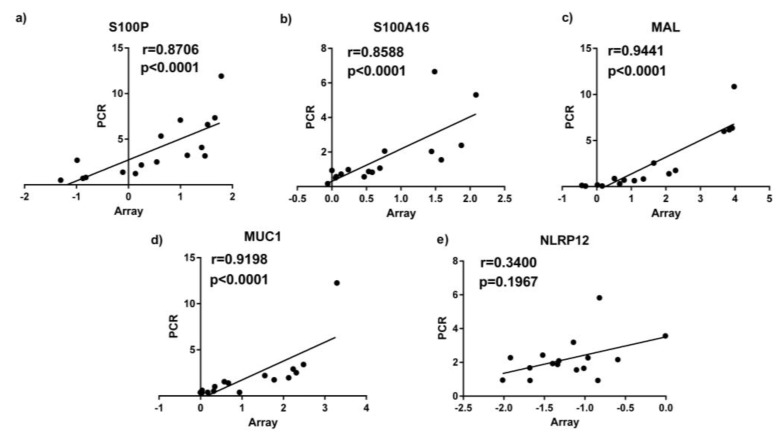
qPCR results correlated with microarray gene expression data (measured by qPCR) in sputum samples. (**a**) S100P; (**b**) S100A16; (**c**) MAL; (**d**) MUC1; (**e**) NLRP12.

Based on the results from PANTHER analysis and their immune functions, we selected 5 genes for PCR follow up in asthmatics, including the S100 calcium binding proteins *S100P*, *S100A16*, cell surface associated mucin 1 (*MUC1*)*,* Nod like receptor pyrin domain 12 (*NLRP12*) and the T cell differentiating protein *MAL* which we consider potentially important in the effect of fat on asthmatic airway inflammation due to their involvement in immune system processes. qPCR results for *S100P, S100A16, MUC1* and *MAL* were highly correlated with the results of microarray (*p* ≤ 0.05; Figure 1).

**Table 3:** 25 Genes involved in the immune system process with their gene functions, fold change and direction of change in regulation in sputum from asthmatics at 4 h after a high fat meal compared with baseline.

Accession	Gene Symbol	Gene Name	Fold Change	↑/↓	Gene Function
NM_002371.2	MAL	T-cell differentiation protein	2.74	↑	Lipid binding peptidase activator activity involved in apoptotic processes
NM_005656.2	TMPRSS2	Transmembrane protease, serine 2	2.56	↑	Protein binding
NM_004433.3	ELF3	E74-like factor 3	2.09	↑	RNA polymerase II core promoter proximal region sequence-specific DNA binding
NM_182502.2	TMPRSS11B	Transmembrane protease, serine 11B	1.95	↑	Serine-type endopeptidase/peptidase activity
NM_144670.2	A2ML1	Alpha-2-macroglobulin-like 1	1.94	↑	Peptidase activity;cytokine activity; serine-type endopeptidase inhibitor activity
NM_012431.1	SEMA3E	Sema domain, immunoglobulin domain (Ig), short basic domain, secreted	1.93	↑	Receptor binding
NM_080388.1	S100A16	S100 calcium binding protein A16	1.86	↑	Novel calcium-binding signalling protein of dietary
NM_001093771.1	TXNRD1	Thioredoxin reductase 1	1.76	↑	Thioredoxin-disulfide reductase activity
NM_005980.2	S100P	S100 calcium binding protein P	1.74	↑	Calcium ion binding; receptor binding; signal transduction
NM_006238.2	PPARD	Peroxisome proliferative activated receptor, delta	1.52	↑	NF-kappaB binding
NM_144687.1	NLRP12	NLR family, pyrin domain containing 12	1.51	↑	Checkpoint of noncanonical NF-κb, inflammtion and tumorigenesis
NM_006613.3	GRAP	GRB2-related adaptor protein	1.55	**↓**	SH3/SH2 adaptor activity
NM_002985.2	CCL5	Chemokine (C-C motif) ligand 5	2.28	**↓**	Chemokine activity
NM_052931.3	SLAMF6	SLAM family member 6	1.90	**↓**	Transmembrane receptor protein tyrosine kinase activity
NM_006120.2	HLA-DMA	Major histocompatibility complex, class II, DM alpha	1.86	**↓**	MHC class II protein complex binding
NM_006725.2	CD6	CD6 molecule	1.85	**↓**	Oxidoreductase activity
NM_178562.2	TSPAN33	Tetraspanin 33	1.83	**↓**	Receptor activity
NM_004454.1	ETV5	Ets variant gene 5	1.80	**↓**	Sequence-specific DNA binding transcription factor activity
NM_016817.2	OAS2	2′-5′-oligoadenylate synthetase 2, 69/71 kDa	1.76	**↓**	Nucleotidyltransferase activity; nucleic acid binding
NM_001040107.1	HVCN1	Hydrogen voltage-gated channel 1	1.7	**↓**	Voltage-gated cation channel activity
NM_005202.1	COL8A2	Collagen, type VIII, alpha 2	1.63	**↓**	Receptor activity
NM_001078.2	VCAM1	Vascular cell adhesion molecule 1	1.63	**↓**	Phosphoprotein phosphatase activity
NM_175571.2	GIMAP8	GTPase, IMAP family member 8	1.58	**↓**	Peptidase inhibitor activity
NM_000732.4	CD3D	CD3d molecule, delta	1.55	**↓**	Protein heterodimerization activity
NM_012463.2	ATP6V0A2	ATPase, H+ transporting, lysosomal V0 subunit a2	1.51	**↓**	Hydrolase activity; cation transmembrane transporter activity; hydrogen ion transmembrane transporter activity

**Table 4:** The differentially expressed genes in sputum from healthy controls at 4 h post-meal compared with baseline with a fold change greater than 1.5 and direction of change in regulation.

Accession	Symbol	Fold Change	↑/↓	Gene Name
NM_002923.1	RGS2	2.42	↑	Regulator of G-protein signalling 2
NM_002274.3	KRT13	2.39	↑	Keratin 13
NM_006317.3	BASP1	1.92	↑	Brain abundant, membrane attached signal protein 1
NM_002965.2	S100A9	1.75	↑	S100 calcium binding protein A9 (calgranulin B)
NM_058172.3	ANTXR2	1.71	↑	Anthrax toxin receptor 2
NM_020226.3	PRDM8	1.69	↑	PR domain containing 8
NM_004913.2	C16orf7	1.62	↑	Chromosome 16 open reading frame 7
NM_003143.1	SSBP1	1.55	↑	Single-stranded DNA binding protein 1
NM_024419.3	PGS1	1.53	↑	Phosphatidylglycerophosphate synthase 1
NM_152361.1	EID2B	1.52	↑	EP300 interacting inhibitor of differentiation 2B
NM_015104.1	ATG2A	1.51	↑	ATG2 autophagy related 2 homolog A
NM_015352.1	POFUT1	1.51	↑	Protein O-fucosyltransferase 1
NM_002443.2	MSMB	1.50	↑	Microseminoprotein, beta
NM_017585.2	SLC2A6	1.51	↓	Solute carrier family 2, member 6

**Figure 2:**
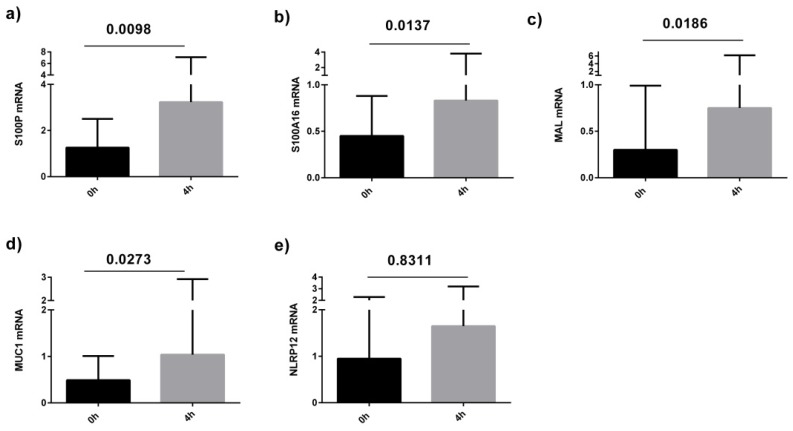
Level of sputum cell gene expression (measured by qPCR) in asthmatics at baseline and 4 h after a high fat meal; (**a**) S100P; (**b**) S100A16; (**c**) MAL; (**d**) MUC1; (**e**) NLRP12.

**Figure 3:**
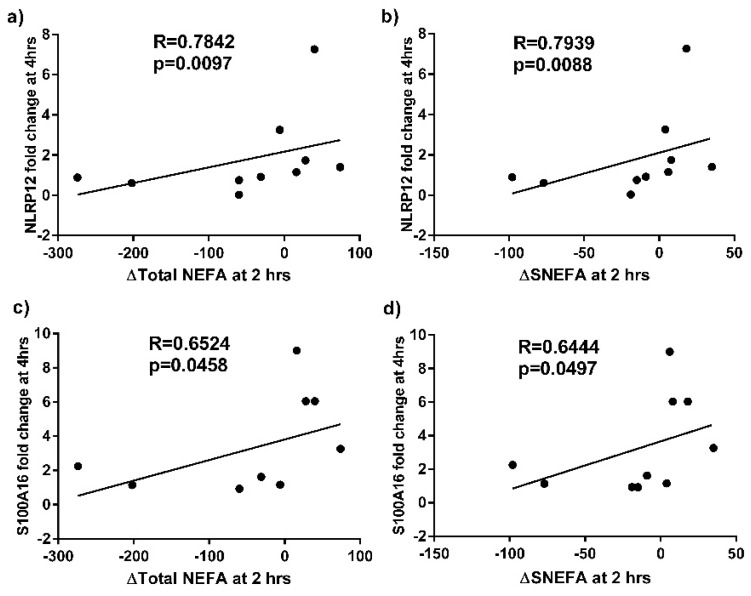
Correlations between the fold change in sputum cell NLRP12 gene expression at 4 h *vs.* baseline and (**a**) change in (∆) total non-esterified fatty acids (NEFA)(mg/L) at 2 h *vs.* baseline; and (**b**) ∆saturated NEFA (sNEFA)(mg/L) at 2 h *vs.* baseline, in subjects with asthma. Correlations between the fold change in sputum cell S100A16 gene expression at 4 h *vs.* baseline and (**c**) ∆Total NEFA (mg/L) at 2 h *vs.* baseline; and (**d**) ∆sNEFA (mg/L) at 2 h *vs.* baseline, in subjects with asthma.

qPCR analysis was performed on sputum samples from 11 asthmatics before and after the high fat meal. Confirming the microarray analysis, we determined that *S100P, S100A16, MAL* and *MUC1* were significantly increased in the asthma group at 4 h post-meal compared with baseline (Figure 2). We also observed a correlation between the fold change in sputum cell *NLRP12* and *S100A16* gene expression at 4 h compared to baseline and the change in total and saturated non-esterified plasma fatty acid concentrations at 2 h compared to baseline (Figure 3).

## 4. Discussion

The present study used microarray technology to investigate changes in the gene expression profile of airway cells in asthma that drive airway inflammation following a high-fat mixed meal in asthma. We have previously demonstrated that a high fat intake increases neutrophilic airway inflammation in subjects with asthma [13]; however, we have a limited understanding of the molecular mechanisms driving this effect. This study shows differences in gene expression changes due to a high fat meal between asthmatics and healthy controls. In sputum from healthy controls, there were only a small number of changes in gene expression after the high fat meal challenge with only 14 genes altered, whereas, in sputum from asthmatics, 168 genes were differentially expressed after the high fat meal, demonstrating exaggerated responses. In asthmatics, most of the genes that were overrepresented were from immune system processes. Of particular interest, the expression of the alarmins *S100P* and *S100A16*, as well as *MAL* and *MUC1* were significantly increased in the asthmatics at 4 h post-meal.

Alarmins are endogenous molecules that can be released in excess by necrotic cells, stimulated leukocytes or epithelial cells upon tissue injury or infection, promoting activation of innate immune cells through pattern recognition receptors (e.g., TLRs) and stimulating production of proinflammatory mediators such as TNF-α and NFκB. Among the best characterized alarmins are the S100 proteins, which are small, acidic proteins, characterized by distinctive homo- or heterodimeric architecture, with two highly conserved calcium-binding domains [18]. Due to their elevated expression in a broad range of tumor tissues, S100 proteins are considered to be biomarkers for cancer; however, they have previously been shown to exhibit a pro-inflammatory response, involving neutrophil adhesion, migration and release from bone marrow [18]. In this study, we found that the expression of S100P and S100A16 genes were significantly increased in the asthma group at 4 h post-meal compared with baseline.

S100A16 is a novel member of the S100 family, ubiquitously expressed in all human tissues including fat tissues and the lung [19,20] and is associated with a variety of human diseases, such as inflammation disorders, prostate cancer and obesity [21]. The range of physiological functions of S100A16 is largely unknown; however, increased expression of S100A16 has been linked to various detrimental metabolic effects. For example, overexpression of S100A16 in 3T3-L1 preadipocytes has been shown to result in a significant reduction in insulin sensitivity, phosphorylation of AKT and activation of the JNK pathway [22]. S100A16 has also been shown to inhibit osteogenesis by decreasing RUNX2 expression and suppressing MAP kinase signal transduction and ERK1/2 activation [20]. Zhang *et al.* extended these observations, reporting that while S100A16 mediated adipogenesis and reduced insulin sensitivity following a high fat diet, this effect could be inhibited by co-consumption of high calcium levels, which acts to exclude S100A16 from the nucleus, resulting in decreased interaction of S100A16 with p53 [19]. Our study suggests that S100A16 could mediate another detrimental effect of a high fat diet in humans—the migration of neutrophils into the airways. Less is known about the inflammatory effects of S100P, which has been shown to mediate tumor growth, drug resistance and metastasis through RAGE binding on cancer cells [23]. In our PCR data, the increase in S100A16 gene expression post meal was correlated with the increase in total and saturated non-esterified fatty acids in plasma. The increase in S100P and S100A16 gene expression following the high fat meal, suggests that the increased production of these S100 alarmins is contributing to the influx of neutrophils into the airways that we observed following a high fat meal [13].

The NF-κB pathway plays a prominent role in the innate immune response and has previously been shown to be activated by dietary saturated fatty acids, via stimulation of TLR4, resulting in production of pro-inflammatory mediators, such as TNF-α and IL-6 [24,25,26]. Our previous report of increased TLR4 expression in sputum cells after a high fat meal suggests involvement of the NF-κB pathway [13]. The activation of NF-κB is dependent on stimulation of the T cell receptor pathway, which has been shown to be upregulated in this study, with the MUC1 gene being increased at 4 h after a high fat meal. MUC1 is a transmembrane glycoprotein ubiquitously expressed by mucosal epithelial cells and serves a protective function by binding to pathogens [27] and also functioning in a cell signaling capacity [28,29]. MUC1 levels increase in response to inflammatory cytokines such as TNF-α and MUC1 has been reported to have an anti-inflammatory function by suppressing the Toll-like receptor signaling pathway [30]. MUC1 has shown be important in the control of ongoing inflammation driven by a range of airway pathogens [31]. Other evidence of MUC1 as a negative controller of inflammation stems from studies of the inflammatory response to infection. Seongwon *et al.* [31] reported that the level of MUC1 expression was augmented following a *Pseudomonas aeruginosa* infection. We found up-regulation of MUC1 gene in asthmatics after a high fat meal challenge, and this may be a compensatory response, initiated in an attempt to control fat-induced inflammation.

Our data suggest that the NOD-like receptor containing a pyrin domain 12 (NLRP12) gene is also sensitive to metabolic stress, as NLRP12 expression was upregulated following the high fat meal in the microarray data. A similar trend was seen in the PCR analysis, which did not reach statistical significance. NLRP12 is one of the 14 members of the NLRP family, which trigger multiple innate immune effector pathways, including the assembly of inflammasomes. Inflammasomes are large multiprotein platforms that form in response to infection and tissue damage and are responsible for the activation of inflammatory caspases, which induce inflammatory cell death (pyroptosis), maturation, and/or secretion of the pro-inflammatory cytokines including IL-1β and IL-18 [32]. NLRP12 associates with apoptosis-associated speck-like protein containing a carboxy-terminal CARD (ASC) to form an inflammasome and to promote NF-kB activation, when overexpressed [33] and also enhances expression of MHC Class I genes [34]. However, in other contexts, NLRP12 can antagonize TLR and TNF receptor signalling upstream of NF-κB which can suppress inflammatory responses [35]. Thus, dependent on the context and cell type, NLRP12 either promotes or antagonizes immune and inflammatory responses, which has also been observed for several other NLRPs [36]. Another of the NLRP family, NLRP3, has been shown to sense metabolic stress signals, including high ATP [37] and glucose levels [38] and the presence of saturated fatty acids [39]. Lipotoxic levels of the saturated fatty acid, palmitate, activate NLRP3, and, accordingly, NLRP3 knockout mice are protected against accumulation of fatty acids in the liver and development of hepatic steatosis [40]. In our PCR data, the increase in NLRP12 gene expression post meal was strongly correlated with the increase in total and saturated non-esterified fatty acids in plasma, suggesting that NLRP12 is also sensitive to high fatty acid levels and may be contributing to the airway inflammatory response via NF-κB activation.

Myelin and lymphocyte protein (MAL) is a hydrophobic membrane proteolipid. MAL can be found in T-cells, some epithelial cells and the nervous system [41]. MAL has been localized to the endoplasmic reticulum of T-cells and is reported to be a candidate linker protein during T-cell signal transduction, involved in the initiation of antigen specific immune responses [42]. Polyunsaturated fatty acids (PUFAs) have previously been shown to modify T cell function, independent of eicosanoids production [43]. Our study suggests that a high fat load increases MAL gene expression in asthma, a gene which could be modifying T-cell signal transduction, thereby inducing airway inflammation.

Several limitations of this study need to be taken into consideration. The high fat mixed meal (two breakfast burgers and two hash browns) contained a mixture of saturated fat, omega-6 polyunsaturated fat and carbohydrate. Therefore, it is not possible to establish which macro- or micronutrients contributed to the activation of inflammatory pathways in asthmatics. However, the correlations that we observed between the change in total and saturated NEFA and fold change in gene expression of NLRP12 and S100A16, as well as the correlations that we have previously observed between changes in total and saturated fatty acids and changes in inflammatory cells [13], indicate that dietary saturated fat is contributing to the observed effects. Further studies are planned to examine the effects of dietary fat *versus* carbohydrate on airway inflammation in asthma and also to examine the effects of different types of fatty acids. Secondly, the sample size of the current study is relatively small. Nonetheless, in this group of subjects, who were matched for demographic characteristics, we had an adequate sample size to identify significant differences. Thirdly, the time frame of the study is short; however, we believe that the study design is biologically relevant, as many studies have shown that postprandial inflammation occurs following consumption of meals high in refined carbohydrates and fat [44]. This occurs via mechanisms such as activation of innate immune receptors, which sense nutrient excess, initiating an immune response within 1–4 h, demonstrating that these systems are in dynamic interplay and can be rapidly influenced by external stimuli. Future work is required to examine the long term effects of high fat intake on molecular mechanisms of airway inflammation. Lastly, this study presents gene expression data; however, future studies should follow up protein levels. We are unable to measure associated protein levels in this study, as we were unable to validate ELISA methods to allow measurement in sputum supernatant due to the effects of Dithiothreitol (DTT), and we do not have other appropriate samples stored in this sample set. Finally, sputum samples are, by nature, a heterogenous mixture of inflammatory cells. Further studies are required to understand the effects of diet on activity and programming of individual cell types.

## 5. Conclusions

Currently available asthma treatments are not effective in preventing airway inflammation and maintaining asthma control in many individuals. Our data identifies several genes that contribute to neutrophilic airway inflammation following consumption of a high fat meal in asthmatics, which may prove to be useful therapeutic targets for immunomodulation. While dietary fat restriction would also provide a means of reducing fat-induced inflammation, targeting these genes may provide an alternative approach for some asthmatics, particularly those who are obese and are habitually consuming diets with a high and undesirable fat content.
